# Association of long-term visit-to-visit variability of HbA1c and fasting glycemia with hypoglycemia in type 2 diabetes mellitus

**DOI:** 10.3389/fendo.2022.975468

**Published:** 2022-08-11

**Authors:** Chen Long, Yaling Tang, Jiangsheng Huang, Suo Liu, Zhenhua Xing

**Affiliations:** ^1^ Department of General Surgery, The Second Xiangya Hospital, Central South University, Changsha, China; ^2^ Department of Cardiothoracic Surgery, The Third Xiangya Hospital of Central South University, Changsha, China; ^3^ Department of Emergency Medicine, The Second Xiangya Hospital, Central South University, Changsha, China

**Keywords:** Severe hypoglycemia, HbA1c variability, fasting glycemia variability, type 2 diabetes mellitus, comparative analysis

## Abstract

**Background:**

Self-management of blood glucose levels to avoid hypoglycemia is vital for patients with type 2 diabetes mellitus (T2DM). The association between specific metrics of glycemic variability (glycosylated hemoglobin A1c [HbA1c] and fasting plasma glucose [FPG]) and severe hypoglycemia has not been fully studied in patients with T2DM.

**Methods:**

In this *post hoc* analysis, patients with established T2DM with a high risk of cardiovascular disease were included in the Action to Control Cardiovascular Risk in Diabetes (ACCORD) study. The Cox proportional hazards model was used to investigate the relationship between glycemic variability and hypoglycemia requiring medical assistance (HMA) and hypoglycemia requiring any third-party assistance (HAA). The prognostic value of HbA1c/FPG variability for our predefined outcomes was compared using Harrell’s C method.

**Results:**

After adjusting for confounders, each increase in HbA1c variability of 1 standard deviation (SD) indicated a higher risk of HAA (hazard ratio [HR]: 1.10; 95% confidence interval [CI]: 1.03–1.16; P < 0.01) and HMA events (HR: 1.11; 95% CI: 1.03–1.20; P < 0.01). Meanwhile, each increase in FPG variability of 1 SD increased the risk of HAA (HR: 1.40; 95% CI: 1.31–1.49; P < 0.01) and HMA events (HR: 1.46; 95% CI: 1.35–1.57; P < 0.01). Meanwhile, models, including FPG variability, had better prognostic value for our predefined outcomes than HbA1c variability (P < 0.01).

**Conclusions:**

Increased visit-to-visit variability in HbA1c and fasting glycemia is associated with a greater risk of severe hypoglycemic events in T2DM patients. FPG variability is a more sensitive indicator than HbA1c variability.

**Trial registration:**

http://www.clinicaltrials.gov. Unique identifier: NCT00000620.

## Introduction

Type 2 diabetes mellitus (T2DM) is a major public health challenge that requires personalized lifetime management. Glucose-lowering therapy is essential to prevent T2DM progression (1, 2). The dose of glucose-lowering medications, such as insulin and metformin, are adjusted according to the monitored glucose level, which is fundamental in the management of T2DM patients. Severe hypoglycemia is defined as episodes of low plasma glucose concentration in T2DM patients receiving glucose-lowering medication, which exposes the patient to potential harm, such as confusion, disorientation, convulsion, loss of consciousness, major adverse cardiac events, and even death (3). Understanding the risk factors to immediately identify severe hypoglycemia may assist health providers, caregivers, and specialists in developing up-to-date diabetes management strategies for T2DM patients, especially those at high risk of cardiovascular disease.

The DEVOTE study demonstrated that day-to-day fasting plasma glucose (FPG) variability is associated with severe hypoglycemia (4). However, all participants in the DEVOTE study received insulin treatment only but without other oral medication, which limits its generalizability. Furthermore, there are several metrics for the assessment of glycemic variability in different settings. FPG variability is a long-term metric based on visit-to-visit FPG measurements (4, 5). Glycosylated hemoglobin A1c (HbA1c) is also a common metric to evaluate the long-term status of glycemia. The DEVOTE study only focused on the relationship between day-to-day FPG variability and the risk of severe hypoglycemia. Few studies have compared the efficacy of different metrics in predicting undesirable outcomes and explored the associations between long-term HbA1c variability and severe hypoglycemic events, ignoring the predictive role of HbA1c variability. Therefore, this *post-hoc* analysis aimed to determine the association between HbA1c/FPG variability and severe hypoglycemic events, as well as comparing the predictive efficacy between FPG variability and HbA1c variability.

## Method

### Study design

We conducted a *post-hoc* analysis of data from the Action to Control Cardiovascular Risk in Diabetes (ACCORD) study, a randomized trial involving 10,251 volunteers with T2DM from 77 clinical sites in the U.S and Canada. The detailed design and principal results have been described previously (6, 7). The ACCORD trial was a double 2×2 factorial trial that primarily investigated whether intensified control of blood glucose (HbA1c, <7.0% vs. 7.0%–7.9%), blood pressure (systolic blood pressure [SBP], <120 vs. <140 mmHg), or lipids (placebo vs. fenofibrate) could reduce the incidence of cardiovascular disease (CVD) in T2DM patients with CVD or high CVD risk. Among the enrolled patients in the ACCORD trial, 10,052 fulfilled the inclusion criteria for HbA1c variability analysis and 10,068 for FPG variability analysis. Patients with less than three measurements of HbA1c/FPG were excluded from our analysis. Included patients were then primarily randomized into two groups based on their glucose-lowering therapy: intensive group and standard group. They were then assessed every 4 months, including HbA1c/FPG measurements.

### Measures of HbA1c/FPG variability

HbA1c/FPG levels were measured at a core laboratory every 4 months. HbA1c/FPG variability was defined as the intra-individual variability in HbA1c/FPG between visits using average successive variability (ASV), defined as the average absolute difference between successive values.

### Study outcomes

The occurrence of severe hypoglycemic events was the primary outcome. The researcher reiterated the course instructions every visit on how to recognize, prevent, and self-treat hypoglycemia. Severe hypoglycemia was defined as plasma glucose below 50 mg/dL (2.8 mmol/L) or presence of symptoms that promptly resolved with oral carbohydrate, intravenous glucose, or parenteral glucagon. Severe hypoglycemic events were recorded in detail at each visit. We defined two types of severe hypoglycemia events in this study, hypoglycemia requiring medical assistance (HMA) and hypoglycemia requiring any third-party assistance (HAA), as described previously. Generally, if participants had episodes requiring hospitalization or emergency medical care, they were defined as HMA. Similarly, if participants had episodes requiring assistance from any person or organization, regardless of whether they are medical or non-medical, it was defined as HAA.

### Statistical analysis

The baseline characteristics of the included patients are presented as mean ± standard deviation (SD) with normal distribution or as median (25th and 75th percentiles) with skewed distribution. Continuous variables were compared using a Student’s t-test or Mann–Whitney U test according to the distribution type, and categorical variables were compared using a chi-square test. First, the participants were divided into four groups based on mean HbA1c/FPG levels. Then, for each group, they were further divided into two groups based on high variability (greater than or equal to the median) or low variability (below the median) for the comparative study to analyze the risk of HAA/HMA events. Cox proportional hazards models assessed the relationship between the ASV of HbA1c/FPG and severe hypoglycemic events. Model 1 was adjusted for mean FPG and HbA1c levels, age, sex, race, and glucose control strategy (intensive/standard). Model 2 was adjusted for model 1 in addition to a history of CVD, education, depression (yes/no), cigarette smoking (current or not), diabetes duration, alcohol consumption (mean days per week), body mass index, low-density lipoprotein, high-density lipoprotein, and glomerular filtration rate. Restricted cubic splines with four knots in the 5th, 35th, 65th, and 95th percentiles were used to explore the nonlinear association between the ASV of HbA1c/FPG and our predefined severe hypoglycemia events. We found no evidence of violation of the proportional hazard assumption based on tests using Schoenfeld residuals. Discrimination in the models was assessed using Harrell’s C-statistic. The prognostic value of ASV of HbA1c/FPG for our predefined outcomes was compared. Sensitivity analysis was performed to verify the robustness of our results. First, to avoid bias in the Cox proportional hazards models, we used competing risk models (modeling sub-distributional hazard ratios) for our predefined outcomes in the sensitivity analysis. Then, we applied competing event of all-cause mortality as a permanent condition to prevent the occurrence of our predefined outcomes. Next, we conducted interaction and stratified analyses considering age (<75 and ≥75 years), sex, race, duration of diabetes (≥10 or <10 years), and glucose control strategy (intensive or standard). All statistical analyses were two-sided, and a P-value of <0.05 was considered statistically significant. All analyses were performed using STATA17.0 software purchased from StataCorp LLC (College Station, TX, USA).

## Result

### Patient characteristics

**Figure 1:**
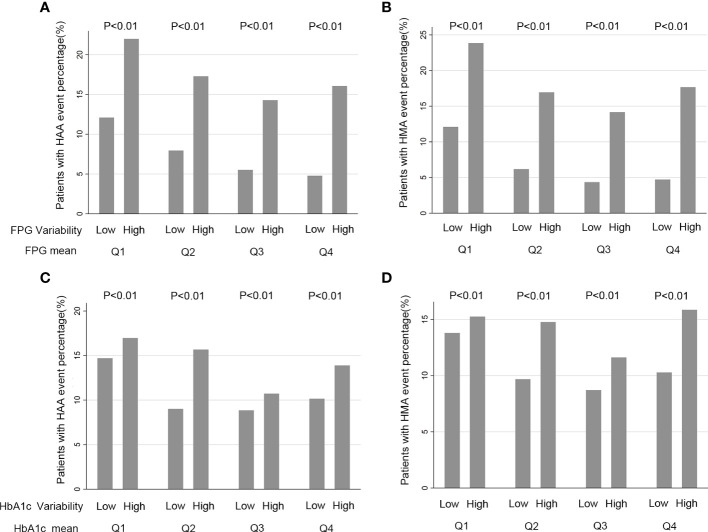
Quartiles of HbA1c/FPG variability and rate HAA/HMA Events a Function of Baseline HbA1c/FPG. **(A, B)** FPG (Fasting Plasma Glucose) and FPG variability, Q1 is defined as a FPG mean located in first quartile, Q2 located a FPG mean in second quartile, Q3 a FPG mean located in third quartile and Q4 a FPG mean located in a fourth quartile; **(C, D)** HbA1c (hemoglobin A1c) and HbA1c variability. Q1 is defined as a HbA1c mean located in first quartile, Q2 a HbA1c mean located in second quartile, Q3 a HbA1c mean located in third quartile and Q4 a HbA1c mean located in fourth quartile.

The baseline characteristics of the included participants are presented in **Table 1** based on HbA1c/FPG variability (below the median versus greater than or equal to the median). The median number of HbA1c measurements was 14 (interquartile range [IQR]: 11–17) and FPG measurement was 11 (IQR: 8–16) (**Supplementary Figures S1, S2**). The median HbA1c and FPG variability were 0.55 ± 0.29 and 30.9 ± 17.5, respectively (**Supplementary Figures S3, S4**). The mean HbA1c and FPG during the follow-up period were 7.4% ± 0.86% and 144.6 ± 33.9 mg/dL, respectively. Patients with standard glucose control had a higher HbA1c/FPG variability. Additionally, the high variability group had higher rates of occurrence of HAA/HMA events in the Q1 to Q4 quartiles of mean HbA1c/FPG levels (**Figure 1**).

**Table 1:** Baseline characteristics of included participants.

	Low HbA1c variability	High HbA1c variability	P-value	Low FPG variability	High FPG variability	P-value
**N**	5026	5026		5034	5034	
**Age, mean ± SD; yr**	63.6 ± 6.7	62.0 ± 6.5	<0.001	63.1 ± 6.6	62.5 ± 6.7	<0.001
**Female**	1923 (38.3%)	1933 (38.5%)	0.837	1897 (37.7%)	1966 (39.1%)	0.157
**Race**			<0.001			<0.001
**No-White**	1767 (35.2%)	2004 (39.9%)		1723 (34.2%)	2057 (40.9%)	
**White**	3259 (64.8%)	3022 (60.1%)		3311 (65.8%)	2977 (59.1%)	
**Glycemic control**			<0.001			<0.001
**Standard**	2849 (56.7%)	2176 (43.3%)		3018 (60.0%)	2017 (40.1%)	
**Intensive**	2177 (43.3%)	2850 (56.7%)		2016 (40.0%)	3017 (59.9%)	
**History of CVD**	1705 (33.9%)	1811 (36.0%)	0.027	1648 (32.7%)	1879 (37.3%)	<0.001
**Education**			<0.001			<0.001
**Less than high school**	635 (12.6%)	829 (16.5%)		645 (12.8%)	824 (16.4%)	
**High school graduate**	1316 (26.2%)	1346 (26.8%)		1341 (26.7%)	1322 (26.3%)	
**Some college**	1646 (32.8%)	1652 (32.9%)		1657 (32.9%)	1648 (32.8%)	
**College graduate or more**	1425 (28.4%)	1197 (23.8%)		1388 (27.6%)	1237 (24.6%)	
**Proteinuria**	953 (19.0%)	1041 (20.7%)	0.028	898 (17.8%)	1101 (21.9%)	<0.001
**History of heart failure**	226 (4.5%)	248 (4.9%)	0.302	189 (3.8%)	287 (5.7%)	<0.001
**Depression**	1089 (21.7%)	1281 (25.5%)	<0.001	1095 (21.8%)	1281 (25.5%)	<0.001
**Duration of diabetes, mean ± SD; yr**	11.2 ± 7.7	10.4 ± 7.4	<0.001	9.7 ± 7.2	11.9 ± 7.8	<0.001
**Current smoker**	617 (12.3%)	775 (15.4%)	<0.001	626 (12.4%)	770 (15.3%)	<0.001
**Alcohol/week, mean ± SD; times**	1.1 ± 2.9	0.9 ± 2.5	<0.001	1.1 ± 2.9	0.9 ± 2.5	<0.001
**BMI, mean ± SD; kg/m^2^**	31.7 ± 5.3	32.7 ± 5.4	<0.001	32.2 ± 5.3	32.3 ± 5.5	0.184
**Cholesterol, mean ± SD; mg/dL**	180.4 ± 39.7	186.3 ± 43.6	<0.001	181.5 ± 40.3	185.2 ± 43.2	<0.001
**Triglyceride, mean ± SD; mg/dL**	174.9 ± 115.6	205.9 ± 174.7	<0.001	186.0 ± 124.8	194.7 ± 169.5	0.004
**LDL, mean ± SD; mg/dL**	103.6 ± 32.7	106.2 ± 34.9	<0.001	103.8 ± 33.0	106.1 ± 34.7	<0.001
**HDL, mean ± SD; mg/dL**	42.7 ± 11.9	41.0 ± 11.3	<0.001	41.7 ± 11.2	42.1 ± 12.0	0.118
**SBP, mean ± SD; mmHg**	135.8 ± 16.6	136.8 ± 17.5	0.004	135.7 ± 16.7	137.0 ± 17.4	<0.001
**DBP, mean ± SD; mmHg**	73.9 ± 10.4	75.8 ± 10.8	<0.001	74.9 ± 10.4	74.8 ± 10.9	0.548
**HR, HR, mean ± SD; bpm**	71.7 ± 11.6	73.6 ± 11.8	<0.001	72.0 ± 11.6	73.3 ± 11.9	<0.001
**GFR, mean ± SD; ml/min/1.73 m2)**	89.8 ± 26.3	92.3 ± 27.9	<0.001	91.6 ± 28.4	90.5 ± 25.8	0.054

HbA1c, glycated hemoglobin A1c; FPG, Fasting plasma glucose; SD, standard deviation; CVD, cardiovascular disease; LDL, Low density lipoprotein; HDL, high density lipoprotein; SBP, systolic blood pressure; DBP, diastolic blood pressure; HR, heart rate; GFR, glomerular filtration rate.

### HbA1c/FPG variability and hypoglycemia

For the analysis of HbA1c variability, the patients were grouped into four quartiles based on HbA1c/FPG variability. Considering the lowest HbA1c variability quartile (Q1) as a reference, Q2 had an increase in the risk of HAA events by 19%, Q3 by 37%, and Q4 by 42%. Furthermore, Q2 had an increase in the risk of HMA events of 21%, Q3 by 33%, and Q4 by 57% in the fully adjusted model 2 (**Table 2**). Similarly, for FPG variability, Q2 had an increase in the risk of HAA events of 17%, Q3 by 48%, and Q4 by 120%, whereas Q2 had an increase in the risk of HMA events of 18%, Q3 by 65%, and Q4 by 141% in the fully adjusted model 2 (**Table 2**).

**Table 2:** Association between HbA1c/FPG variability and predefined outcomes.

	Incidence rate per 1000 person years	HR (95%CI)	
		Model 1	Model 2
**HbA1c variability**			
**HAA**			
**Q1**	21.4	Ref	Ref
**Q2**	26.4	1.22(1.03-1.45)	1.19(1.00-1.42)
**Q3**	29.7	1.45(1.17-1.64)	1.37(1.16-1.63)
**Q4**	32.9	1.36(1.13-1.63)	1.42(1.18-1.71)
**P for trend**		<0.01*	<0.01*
**Per SD increase**		1.06(1.00-1.13)	1.09(1.03-1.16)
**HMA**			
**Q1**	13.2	Ref	Ref
**Q2**	16.4	1.23(0.99-1.52)	1.21(0.97-1.50)
**Q3**	19.2	1.43(1.16-1.76)	1.33(1.14-1.74)
**Q4**	23.1	1.49(1.19-1.86)	1.57(1.25-1.97)
**P for trend**		<0.01*	<0.01*
**Per SD increase**		1.07(1.00-1.15)	1.10(1.02-1.19)
**FPG variability**			
**HAA**			
**Q1**	17.1	Ref	Ref
**Q2**	22.1	1.24(1.02-1.50)	1.17(0.97-1.42)
**Q3**	29.2	1.64(1.36-1.97)	1.48(1.23-1.78)
**Q4**	42.4	2.51(2.08-3.04)	2.20(1.81-2.67)
**P for trend**		<0.01*	<0.01*
**Per SD increase**		1.49(1.40-1.58)	1.42(1.33-1.51)
**HMA**			
**Q1**	10.3	Ref	Ref
**Q2**	13.2	1.22(0.96-1.55)	1.18(0.92-1.50)
**Q3**	19.6	1.81(1.44-2.27)	1.65(1.31-2.08)
**Q4**	28.9	2.73(2.16-3.47)	2.41(1.89-3.07)
**P for trend**		<0.01*	<0.01*
**Per SD increase**		1.55(1.44-1.67)	1.47(1.37-1.59)

*P value<0.05

Model 1: FPG, HbA1c, age, sex, race, glucose control strategy.

Model 2: FPG, HbA1c, age, sex, race, glucose control strategy, history of cardiovascular disease, education, depression, cigarette, duration of diabetes, alcohol, body mass index, low-density lipoprotein, high-density lipoprotein, glomerular filtration rate.

HbA1c, glycated hemoglobin A1c; FPG, Fasting plasma glucose; HR, hazard risk; CI, confidence interval; SD, standard deviation; HAA, hypoglycemia requiring any third-party assistance; HMA, hypoglycemia requiring medical assistance.

When HbA1c/FPG variability was used as a continuous variable in model 2, each increase in HbA1c variability of 1 SD increased the risk of HAA [hazard ratio (HR): 1.10; 95% confidence interval (CI), 1.03–1.16; P < 0.01) and HMA events (HR: 1.11; 95% CI: 1.03–1.20; P < 0.01; model 2). Meanwhile, each increase in FPG variability of 1 SD increased the risk of HAA (HR: 1.40; 95% CI: 1.31–1.49; P < 0.01; model 2) and HMA events (HR: 1.46; 95% CI: 1.35–1.57; P < 0.01).

**Figure 2:**
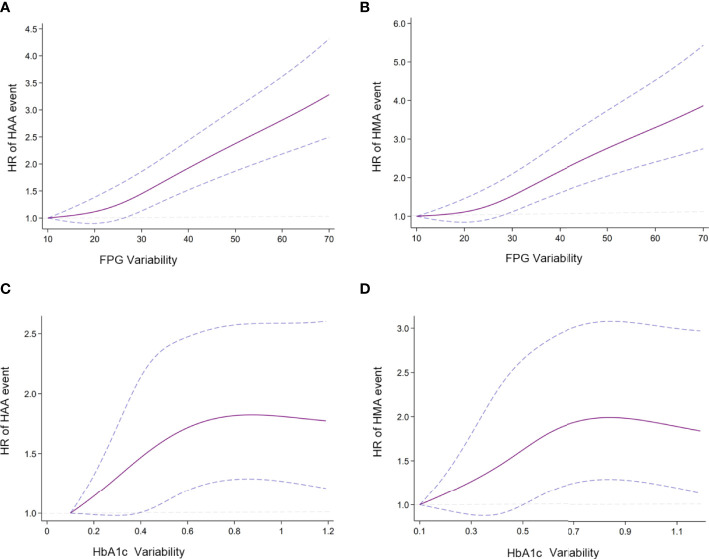
Association of predicted HAA/HMA event and HbA1c/FPG variability **(A)** Association of FPG variability and HAA event; **(B)** Association of FPG variability and HMA event; **(C)** Association of HbA1c variability and HAA events; **(D)** Association of HbA1c variability and HMA event. Hazard ratios are indicated by solid lines and 95% CIs by areas between two dotted lines. (Reference point is the lowest value for each curve) The Reference knots were placed at the 5th, 35th, 65th, 95th, centiles of HbA1c/FPG variability distribution. HbA1c/FPG variability were adjusted using model 2.

In **Figure 2**, we used restricted cubic splines to flexibly model and visualize the relationship between FPG/HbA1c variability and our predefined endpoints. This illustrated that the risk of HAA/HMA events increased sharply with the increase in FPG variability without any plateau period (**Figures 2A, B**). However, the risk of HAA/HMA did not increase and reached a plateau period, when HbA1c variability exceeded 0.7 (**Figures 2C, D**).

### Sensitivity and subgroup analysis

**Figure 3:**
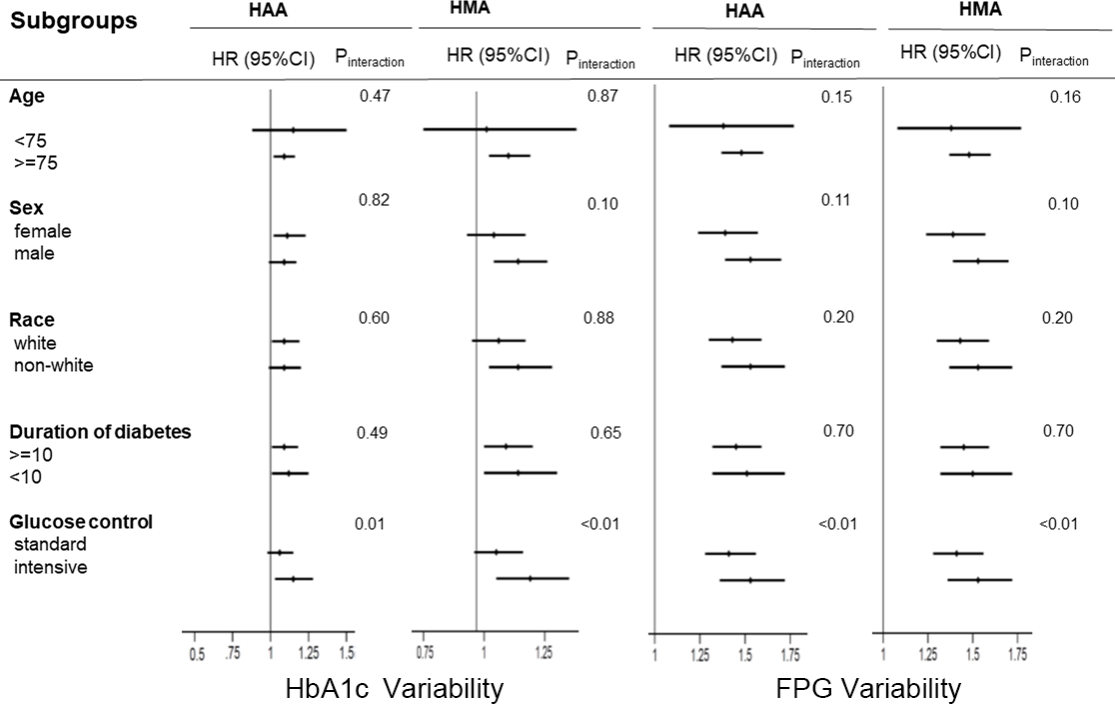
Hazard ratios per one standard deviation increase in the HbA1c/FPG variability for the predefined endpoints. Each stratification was adjusted for all factors in model 2 (fasting plasma glucose, plasma glucose control strategy, age, race, female, history of cardiovascular disease, education, depression, cigarette, duration of diabetes, alcohol, body mass index, low-density lipoprotein, high-density lipoprotein, glomerular filtration rate, HbA1c), except for the stratification factor itself. HAA, hypoglycemia requiring any third-party assistance; HMA, hypoglycemia requiring medical assistance; HbA1c, Hemoglobin A1c; FPG, Fasting plasma glucose.

We used the competing risk model instead of the Cox proportional risk model in sensitivity analysis, which yielded consistent results (**Supplementary Table 1**). Furthermore, subgroup analysis results are summarized in **Figure 3**. The results showed that no factor played an interactive role in the association between HbA1c variability and HAA/HMA events, except glucose control therapy, suggesting a stronger effect of HbA1c variability on the risk of HAA/HMA events in patients with intensive glucose control. Similar effects were noted in the association between FPG variability and HAA/HMA events.

### Comparative analysis of predictive efficacy between HbA1c and FPG variabilities

In the comparative analysis, Harrell’s C method was applied to evaluate the predictive efficacy of HbA1c and FPG variabilities in HAA/HMA events. **Table 3** shows that FPG variability was more powerful in predicting HAA/HMA events than HbA1c variability in both adjusted models 1 and 2.

**Table 3:** Difference of Harrell’s C index between HbA1c variability and FPG variability in predicting HAA/HMA.

	Model 1	P value	Model 2	P value
**HAA**				
HbA1c variability	0.6816(0.6606-0.7025)	<0.01*	0.7078(0.6881-0.7274)	<0.01*
FPG variability	0.6977(0.6773-0.0.7180)	<0.01*	0.7166(0.6972-0.7360)	<0.01*
Difference	0.0161(0.0085-0.02369)	<0.01*	0.0882(0.0037-0.01396)	<0.01*
**HMA**				
HbA1c variability	0.7102(0.7026-0.7495)	<0.01*	0.7243(0.7001-0.7574)	<0.01*
FPG variability	0.7271(0.6847-0.7337)	<0.01*	0.7343(0.7112-0.7575)	<0.01*
Difference	0.0169	<0.01*	0.0101	<0.01*

*P value<0.05.

Model 1: fasting plasma glucose, HbA1c, age, sex, race plasma glucose control strategy.

Model 2: fasting plasma glucose, plasma glucose control strategy, age, race, female, history of cardiovascular disease, education, depression, cigarette, duration of diabetes, alcohol, body mass index, low-density lipoprotein, high-density lipoprotein, glomerular filtration rate, HbA1c.

HbA1c, glycated hemoglobin A1c; FPG, Fasting plasma glucose; HR, hazard risk; CI, confidence interval; SD, standard deviation; HAA, hypoglycemia requiring any third-party assistance; HMA, hypoglycemia requiring medical assistance.

## Discussion

In this *post-hoc* analysis of participants with established T2DM with a high risk of CVD in the ACCORD trial, the two long-term metrics of glycemic variability (HbA1c and FPG) were strongly associated with an increased risk of severe hypoglycemia events, and the associations were independent of mean HbA1c/FPG. Moreover, by comparing the efficacy of these two metrics, we concluded that FPG variability was better than HbA1c variability in predicting both HMA and HAA events. In sensitivity analyses, we found that the glucose control strategy moderated the association between HbA1c/FPG variability and our predefined outcomes, suggesting that patients who received an intensive glucose strategy were more likely to develop hypoglycemia even if they had the same HbA1c/FPG variability.

Both HbA1c variability and FPG variability can be categorized into long-term metrics of glycemic variability because they were obtained through visit-to-visit determinations over a certain period of time (8). Despite there being no comprehensive consensus on clinical significance of glycemic variability, studies showed that FPG variability is associated with increased risk of adverse events and specific diseases, including cardiovascular events, peripheral neuropathy, retinopathy, and arterial stiffness (9–13). Clinical practice and research have focused on HbA1c in T2DM patients, rather than the potential role of HbA1c variability (14–16). Our results suggest that higher HbA1c variability increases the incidence of severe hypoglycemia, indicating that it is important to carefully observe in T2DM patients with higher HbA1c variability and take precautions, even when acceptable levels of HbA1c and FPG are achieved. In the SWITCH Trials, DeVries JH *et al.* concluded that higher day-to-day fasting blood glucose variability is associated with an increased risk of overall symptomatic severe hypoglycemia in insulin-treated patients (5). Our analysis of data from the ACCORD trial also demonstrated that higher long-term visit-to-visit glycemic variability (FPG/HbA1c variability) increased the risk of severe hypoglycemia. Additionally, studies have shown that the choice of glucose control therapy is a potentially modifiable risk factor for hypoglycemia (17–19). Furthermore, previous studies have demonstrated that fasting blood glucose variability is influenced by glucose-lowering medications (5, 20). Our study supported this perspective and found that glucose control therapy played an interactive role in the association between HbA1c/FPG variability and HAA/HMA events in the subgroup analysis (21, 22). Patients receiving standard glucose therapy had higher glycemic variability than those receiving intensive glucose therapy.

Different types of antidiabetic treatments affect glycemic variability and hypoglycemia to various extents in the T2DM population. The ultimate goal for them is to obtain a long-term stable plasma glucose level that has been repeatedly highlighted in various clinical guidelines.

The strengths of this study are that it exploited the data from a large cohort of patients with established T2DM and determined severe hypoglycemic episodes in different glucose-lowering strategies. While a previous study focused on the relationship between a specific single metric of glycemic variability and single severe hypoglycemia event, our study applied multiple metrics (HbA1c/FPG variability) and two types of severe hypoglycemia events (HMA/HAA). To the best of our knowledge, our study is the first to compare the predictive efficacy of HbA1c and FPG using HMA and HAA events.

Severe hypoglycemia is a common complication of T2DM, which is harmful to patient health, with an annual incidence of 0.75–33.8 per 1,000 patients (23–25). Meanwhile, severe hypoglycemia is an distressing experience for these patients with glycemic control therapy that increases the risk of developing physical injuries and emotional burden, affecting quality of life because episodes of severe hypoglycemia could result in cognitive disorientation and loss of consciousness, leading to subsequent accidents and injury, especially in the elderly population (26, 27). The American Diabetes Association working group emphasized that people with diabetes would develop potential harm from all episodes of hypoglycemic events (28). Deleterious effects of hypoglycemia on the cardiovascular system were reported, and multiple mechanisms, including disrupting normal function of blood coagulation and endothelial cells, activating inflammation pathways, and inducing sympathoadrenal response (3). Researchers strive to identify risk factors to predict the occurrence of severe hypoglycemia. Risk factors associated with severe hypoglycemia include age, insulin and sulfonylurea use, and cognitive impairment (29, 30). Increased glycemic variability is a risk factor for severe hypoglycemia. Patients with greater glycemic variability tend to be older, with a long history of diabetes, and less medical compliance, which in turn, leads to a higher incidence of hypoglycemia. (4,5) While there are different metrics representing glycemic variability in various aspects, the association between glycemic variability and severe hypoglycemia remains unclear because of the specific metrics applied in studies and heterogeneity of study design. Moreover, preventing hypoglycemia is prioritized by healthcare providers because it reduces the incidence of cardiac diseases, including cardiac arrhythmia and CVD (31, 32). Future research could shift from emphasizing single metrics to combined glycemic-related metrics to improve the efficacy of predicting hypoglycemic events and reduce their occurrence. The treatment could be various “cocktails” for T2DM patients rather than rigid one, or two cuts-off of metrics based on glycemic-related metrics since changing the glucose therapy would not always adjust all metrics of T2DM patients to an ideal level in a clinical setting.

Our study has some limitations. First, the included patients with CVD or a high risk of CVD had a long duration of T2DM, which put them at a higher risk of hypoglycemia and other adverse events. This may have affected the generalizability of this study, especially in young patients with T2DM with lower CVD risk. Second, the patient characteristics were only measured during inception, without re-evaluation on the follow-up period. Third, all included patients were from the US and Canada and these results may not be applicable to other ethnicities. Another limit to the study is the lack of data on dietary habit and lifestyle modifications, as not all food has the same glycemic index and may be a limitation for FPG/HbA1c evaluation (33).

## Conclusions

T2DM patients with increasing HbA1c and FPG variabilities are at a greater risk of severe hypoglycemia. Additionally, FPG variability is a more sensitive indicator than HbA1c variability. Therefore, clinicians should consider the variability of both HbA1c and FPG as indicators for predicting severe hypoglycemia events and consider them as potential targets for treating patients with T2DM.

## Data availability statement

Publicly available datasets were analyzed in this study. This data can be found here: https://biolincc.nhlbi.nih.gov/home/.

## Ethics statement

The studies involving human participants were reviewed and approved by http://www.clinicaltrials.gov. Unique identifier: NCT00000620). The patients/participants provided their written informed consent to participate in this study. Written informed consent was obtained from the individual(s) for the publication of any potentially identifiable images or data included in this article.

## Author contributions

ZX designed the study and provided methodological expertise. CL and SL drafted the manuscript. CL, SL, YT, JH, and ZX drafted the tables and figures and performed statistical analysis. All authors contributed to the article and approved the submitted version.

## Funding

This work was supported in part by the National Science Foundation of China project 82000298 to ZX.

## Acknowledgments

All authors read, provided critical feedback, and approved the final manuscript. ZX is the guarantor of this work and, as such, had full access to all the data in the study and takes responsibility for the integrity of the data and the accuracy of the data analysis.

## Conflict of interest

The authors declare that the research was conducted in the absence of any commercial or financial relationships that could be construed as a potential conflict of interest.

## Publisher’s note

All claims expressed in this article are solely those of the authors and do not necessarily represent those of their affiliated organizations, or those of the publisher, the editors and the reviewers. Any product that may be evaluated in this article, or claim that may be made by its manufacturer, is not guaranteed or endorsed by the publisher.

## Abbreviations

T2DM, Type-2 diabetes mellitus; FPG, Fasting plasma glucose; HbA1c, Glycosylated hemoglobin A1c; GV, glycemic variability; CVD, Cardiovascular disease; ACCORD, The Action to Control Cardiovascular Risk in Diabetes; SD, Standard deviation; GFR, Glomerular filtration rate; HMA, hypoglycemia requiring medical assistance; HAA, hypoglycemia requiring any third-party assistance.
