# Supplementary material for: Association of long-term visit-to-visit variability of HbA1c and fasting glycemia with hypoglycemia in type 2 diabetes mellitus
Source: Front Endocrinol (Lausanne). 2022 Aug 11;13:975468. doi: 10.3389/fendo.2022.975468 (PMC9402888; doi:10.3389/fendo.2022.975468)
Supplement: Supplementary file 1 [file DataSheet_1.docx]

**Supplementary Appendix**

**Fig S1 Frequency Distribution of Number of HbA1c measurement**


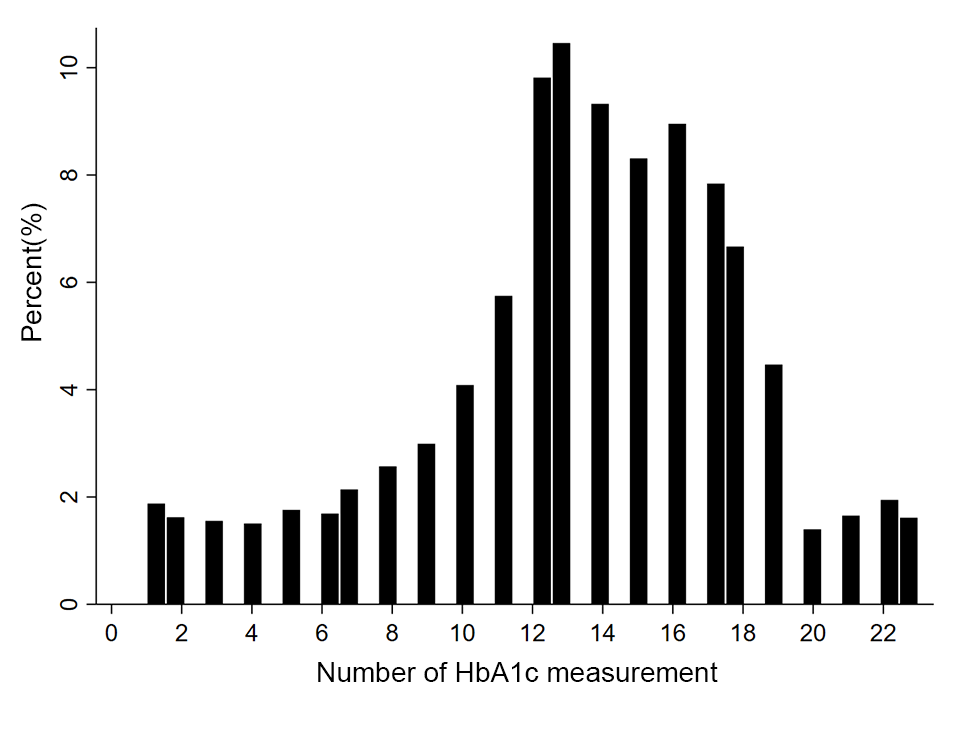
**Fig S2 Frequency Distribution of Number of HbA1c measurement**


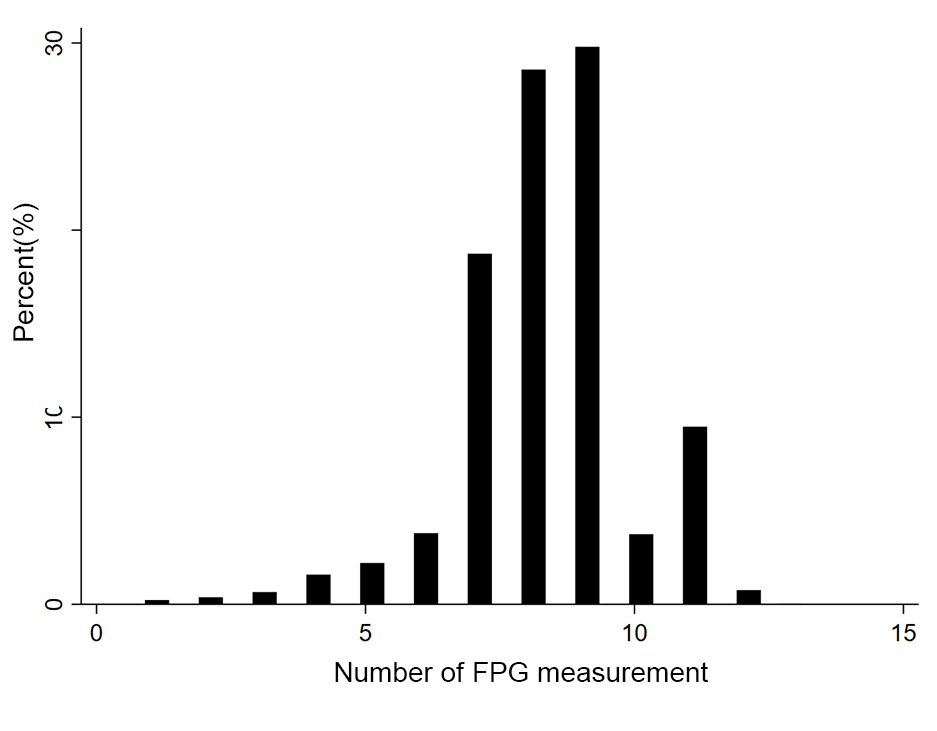


**Fig S3 Frequency Distribution of HbA1c/FPG variability**


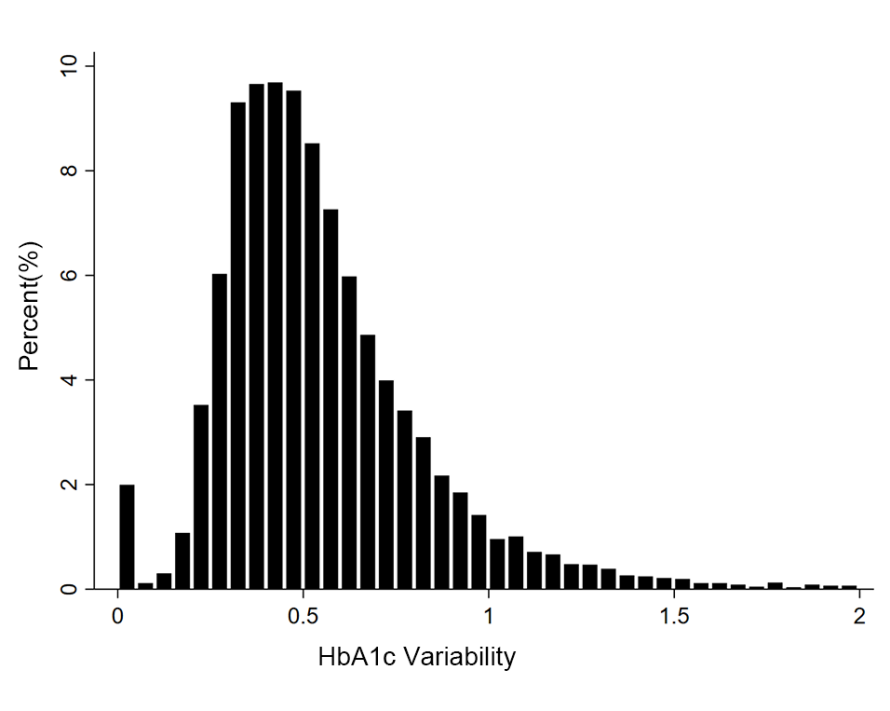


**Fig S4 Frequency Distribution of FPG variability**


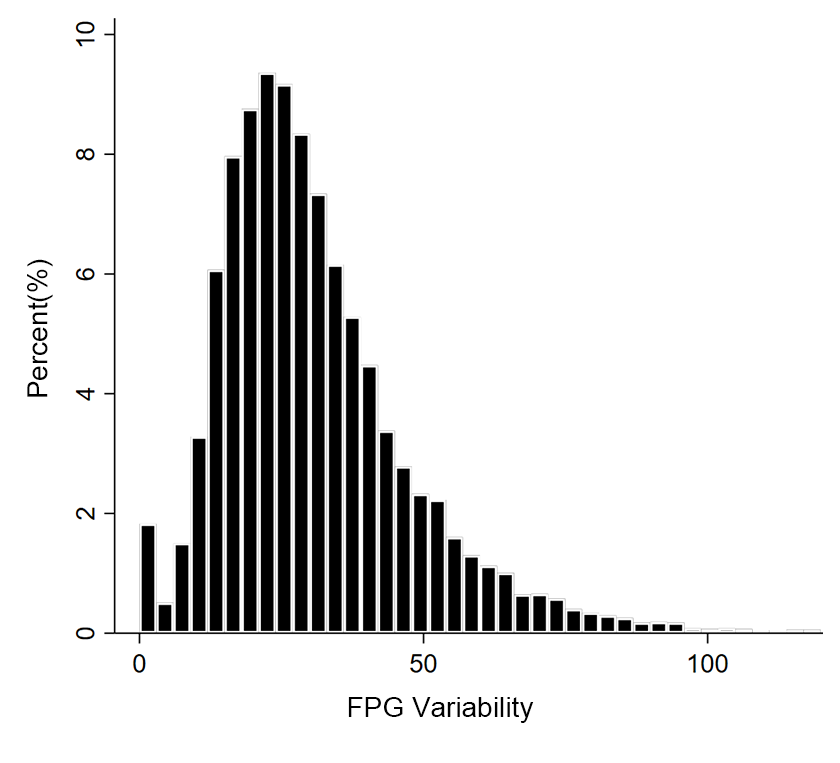


**Table S1 Sensitivity Analysis using competing risk model**

|  | HR (95%CI) | |
| --- | --- | --- |
|  | Model 1 | Model 2 |
| **HbA1C variability** | | |
| **HAA** |  |  |
| Q1 | Ref | Ref |
| Q2 | 1.28(1.07-1.52) | 1.25(1.05-1.48) |
| Q3 | 1.45(1.23-1.72) | 1.43(1.21-1.70) |
| Q4 | 1.41(1.17-1.70) | 1.45(1.20-1.75) |
| **P for trend** | <0.01* | <0.01* |
| **Per SD increase** | 1.07(1.01-1.14) | 1.10(1.03-1.16) |
| **HMA** |  |  |
| Q1 | Ref | Ref |
| Q2 | 1.30(1.05-1.61) | 1.27(1.02-1.57) |
| Q3 | 1.51(1.23-1.87) | 1.48(1.19-1.83) |
| Q4 | 1.56(1.25-1.95) | 1.61(1.28-2.02) |
| **P for trend** | <0.01* | <0.01* |
| **Per SD increase** | 1.09(1.02-1.16) | 1.11(1.03-1.18) |
| **FPG variability** | | |
| **HAA** |  |  |
| 1 | Ref | Ref |
| 2 | 1.29(1.07-1.56) | 1.24(0.97-1.58) |
| 3 | 1.73(1.44-2.07) | 1.76(1.40-2.22) |
| 4 | 2.64(2.19-3.19) | 2.57(2.02-3.27) |
| **P for trend** | <0.01* | <0.01* |
| **Per SD increase** | 1.48(1.39-1.58) | 1.42(1.33-1.51) |
| **HMA** |  |  |
| Q1 | Ref | Ref |
| Q2 | 1.28(1.01-1.62) | 1.27(1.02-1.50) |
| Q3 | 1.92(1.53-2.41) | 1.65(1.31-2.08) |
| Q4 | 2.91(2.30-3.67) | 2.41(1.89-3.07) |
| **P for trend** | <0.01* | <0.01* |
| **Per SD increase** | 1.51(1.43-1.37) | 1.47(1.36-1.59) |

* P value<0.05

Model 1: FPG, HbA1C, age, sex, race, glucose control strategy.

Model 2: FPG, HbA1C, age, sex, race, glucose control strategy, history of cardiovascular disease, education, depression, cigarette, duration of diabetes, alcohol, body mass index, low-density lipoprotein, high-density lipoprotein, glomerular filtration rate.
